# Chronic Diarrhœa

**Published:** 1865-08

**Authors:** George D. Winch

**Affiliations:** Surgeon 42d Wisconsin Vol. Inft.


### CHRONIC DIARRHŒA.

BY GEORGE D. WINCH, M. D.,

*Surgeon 42d Wisconsin Vol. Inft.*

This very frequent disease of the army (by the discharge of troops) will be distributed through many parts of our country. Its frequency and extreme obstinacy should call forth the experience of those who have been previously associated with this disease sufficient to learn its nature and pathology. We will commence with the *post mortem* appearances. The STOMACH, which is described by many as being diminished in size, we have always found of normal bulk, even in those protracted cases where the diminished dimensions is said to be peculiarly frequent. The mucous membrane, generally dark red, seldom softened or contains ulcers. The DUODENUM and JEJUNUM sometimes exhibit appearances of an inflammatory character, but generally nothing decisive. In the ILEUM the change is more noticeable, the whole extent of this is altered, but a gradual increase from the beginning to the ileo-cæcal. valve. The mucous membrane is of a dark or reddish-brown color and thickened. The vessels seemingly injected to their utmost fulness. The solitary follicles and agminated glands participate in the enlargement; but we have not succeeded in finding ulcers. The exhibition of the mucous membrane of the COLON is more conclusive of the true pathology. It is a dark red color, thickened, the solitary follicles enlarged. In some cases there is no ulceration to be found, and I confess I cannot diagnose this class from those in which on examination are found large ragged ulcers. In the first class there will often be extreme pain, the passages very frequent and fetid, also the inflamed membrane seems as susceptible of pressure as the ulcerated one. But in the majority of cases the mucous membrane is ulcerated, disorganization increasing from the beginning to the end. We have seen the whole length of the descending colon present one ragged erosion on its mucous surface, and again the membrane of the whole track of the COLON penetrated by a large number of ulcers from one-half to one inch wide, and one or two inches in length, their edges being raised by a narrow, gray colored lymph deposit.

The fatty LIVER is of common occurrence, especially in cases of long standing, and often the lower portion of the right lobe is so disorganized and softened as to be torn or crushed without force; this last is the effect of congestion, judging from the appearances of this organ in cases of shorter duration. The SPLEEN seldom enlarged, but nearly black and softened, and when increased in size the patient has had the symptoms of remittent fever. KIDNEYS, in some cases, appear normal, in others, enlarged and of a bloodless appearance. Misentric glands enlarged. The pericardium often containing from two to four ounces of fluid.

Causes.—It is an easy task to enumerate many which are known to be common predisposing and exciting causes of this disease; but it is more difficult to trace in each case its own peculiar cause, which would be of great utility both as to therapeutic and dietetic measures. A want of vegetable diet is a common cause, and if not the most frequent, its effects are the most protracted, and difficult to overcome. The patient must necessarily be fed on vegetable diet a sufficient time to change the condition of the vital fluid before a permanent cure can be established. Crowd Poisoning, although no doubt often predisposing, is less frequently the cause of this disease than of camp fevers, and when the patient goes into the hospital or is returned home, he is no more exposed to this loathsome agent. The same can be said of malaria. Occasionally this disease is the continuation of an uncured acute attack.

Symptoms.—Often this disease commences very insiduously, not even awaking the patient to his danger until his case is critical, and then causing no alarm. This class is generally associated with a strong scorbutic taint; there is much debility, both mental and muscular, inattention to surrounding objects, and the condition of his person, passing from two to six liquid stools daily, without pain. Again the patient may be attacked with all the symptoms of acute enteretis, chills followed by fever, frequent and painful discharges, tenderness on pressure, and these symptoms passing gradually into Chronic Diarrhœa. But this last is not of common occurrence. More commonly the patient has symptoms of (commencing) adynamic fever, as slight chills, headache, aching and pain in the limbs, loathing of food, liquid stools, more or less frequent. The countenance is dark, and the icterode hue so often displayed by the malarial influence is frequently present, skin dry and reminds the physician of a convalescent from an eruptive disease. Pulse from 80 to 90, seldom above the latter until late in the disease. In some cases there are rhumatic pains, mostly in the shoulders, and cardiac irritability, producing palpitation. Sometimes there is a craving for food, raw and indigestible vegetables are devoured, doing much injury. This is where the scorbutic taint predominates and a proper vegetable diet is found to be a dietetic measure of great utility. Tongue large and flabby, may present a white or yellowish-white coat, often fissured, but at this stage moist. As the disease advances, emaciation becomes apparent, abdomen flat or concave, and a gurgling is felt and heard by pressure. This gurgling sound can often be produced the whole length of the colon. It is as surely found in the right iliac region as at other points in the large intestine. Pulse becomes more frequent and feeble, but never rises to that frequency so often seen in camp fever, but is very weak. Stools increase in frequency, become dark and fetid, and much tenesmus. In many of these cases that prove fatal, the last three or four days the number of stools and tenesmus will equal those of acute dysentery, and these are the cases where the mucous membrane of the DESCENDING COLON and RECTUM is found disorganized by inflammation and ulceration. No delirium, the mind clear, the patient answering questions till the last. (A strong contrast to camp fever.) The action of the heart grows weaker and weaker until life is extinct. But the patient may recover after all reasonable hope has left the physician. When the pulse is a mere thread in size and no force, by a judicious use of stimulants the vital energies may be brought to that point where proper remedies and nutritious diet will save the patient. A slow and protracted convalescence, with many a relapse of the diarrhœa, must be expected. Recovery is rare where the pulse reaches that extreme feebleness with the adynamic symptoms last described. When it is to take a favorable course the tongue commences cleaning. Where it has been long covered by a dark thick coat, it often commences by throwing off flakes from the posterior and central portions. Pulse stronger and less frequent; skin loses its roughness and becomes moist and of a more natural color. The stools may not decrease in frequency immediately on amendment where they have not passed but from four to six daily, but when they have been frequent and painful they become less frequent and the pain subsides. Urine will at times be small in quantity, but by the proper use of diuretics the patient will pass the normal amount, while its specific gravity will in very rare cases be very light. In a large proportion very slight alkaline reaction, and we have found deposits of urates, oxalates and phosphates.

Treatment.—There is no disease that calls for a more steady and persistent course than Chronic Diarrhœa. Remedies should not be abandoned because of not relieving symptoms in a short time. If he has been exposed to malarial influence he should be removed where he will no longer receive this impairing noxious agent. If the scorbutic taint appears, a judicious and persistent course of vegetable diet should be immediately commenced, with the use of lemonade or citric acid drinks. Oranges, properly used, are grateful and beneficial. The same may be said of apples baked. Animal diet is also necessary to keep the vital powers above that point where the reparable process fails. Beef tea and essence, and chicken soup are the most common and useful articles. Rice in the above preparations is a useful adjunct. Milk must not be neglected, we have found it of great utility where there was not much desire for food, giving very small quantities often. By properly dividing the doses it seldom produces indigestion, and while changing from one article to another of the above preparations, milk can be perseveringly used. The articles of diet should be ordered by the physician, changing them as the circumstanses of the case require. Frequently beef tea, taken with relish, at first, soon produces nausea and vomiting. It is then that some of the other preparations mentioned should be used in its stead. *Never* continue an article after it produces the effect last mentioned. The quantity should also be prescribed.

Mutton chop and soft boiled eggs should be tried, and if not producing indigestion or irritating the bowels, should be continued.

The therapeutic measures of this disease are of far less importance than the dietetic. It is only by a judicious use of tonics, chalybeate astringents, opiates and alcoholic stimulants that benefit can be received. Opiates should be given to quiet pain when there is tormina, in repeated half-grain doses, until it effects its object or when the patient is wakeful and cannot sleep in one grain doses at bed-time to produce sleep, and that should limit the use of anodynes, except by injection, of which we will soon speak. There are many preparations that are lauded, but we will speak of those only which we have used. Tr. Ferri Chlo. in doses of 10 to 15 drops every three hours, is often well borne and very useful in connection with opiates, when they are necessary, or alcoholic stimulants if there is prostration. This has proved a more useful remedy in our hands than Sul. Cupri. Sul. Ferri. The liquor Ferri Pernitratis we have never used, but many Surgeons of much experience with this remedy inform us it is second to none. If any tendency to periodic exacerbations, Quinina should be used. Mineral acids are useful, especially in the scorbutic variety. Give Aromat. Sulph. Acid 10 to 15 gtts. three times daily. If there is hepatic derangement the Nitro-Muri. Acid is more desirable. Tannin, Catechu, and other astringents of that class are of no utility, on the contrary, do injury by irritating the stomach and producing hepatic congestion.

Alcoholic stimulants should be given under the same circumstances as would point to their use in other diseases. They should not be given recklessly; but in cases as described where the system is becoming prostrated below that point where the reparable process can be maintained, alcoholic stimulants should be assiduously employed. Prescribe a certain amount; watch the patient and vary the quantity according to its necessity and effect. *Never* leave this part to the judgment of the nurse. In some cases where depression and prostration are so far advanced as to shut out all reasonable hope of recovery by this course, the patient will commence to mend, and immediately, aided by supporting diet and tonics will soon begin a slow convalescence.

The importance of suitable enemata must be apparent when the pathology of the disease is carefully reflected upon. That the lower portion of the *colon* and *rectum* are generally more seriously diseased than the other parts of that gut, is no longer an uncertainty. That fact clearly indicates their use. Among a list of remedies highly recommended, I much prefer Cupri. Sul. and Tr. Opii, used separately or together according to circumstances. Sul. Cupri. grs. iv., Tr. Opii m. xx Acacia pulv. ʒ ss. Aq. ℥ iv, m., used morning and evening, seems admirably adapted to those cases when the signs and symptoms denote iritation, inflamation and ulceration in the lower portion of the gut. Sulphate Copper will not produce the pain, and will be much longer retained than the Nit. Silver. From these injections, judiciously used, there will be derived much benefit. This class of local applications deserve more notice than is given them. By their use the pain will subside, evacuations become less frequent, and all the local symptoms improve.

To furnish a proper combination of hygenic and dietetic measures, alcoholic stimulants, chalybeate preparations, quinine and local applications, requires much care and attention of the physician.

Nux vomica has been extolled by those who have had experience with the remedy. I have never used it but in that class of cases characterized by no pain, much debility, both bodily and mental, it would, (judging from its action in other cases,) be very beneficial. A large majority of cases will, if judiciously treated, recover although it may require weeks or even months.

